# A new protein binding pocket similarity measure based on comparison of clouds of atoms in 3D: application to ligand prediction

**DOI:** 10.1186/1471-2105-11-99

**Published:** 2010-02-22

**Authors:** Brice Hoffmann, Mikhail Zaslavskiy, Jean-Philippe Vert, Véronique Stoven

**Affiliations:** 1 Mines ParisTech, Centre for Computational Biology, 35 rue Saint Honore, Fontainbleau F-77300, France; 2 Centre for Mathematical Morphology, 35 rue Saint Honore, Fontainebleau F-77300, France; 3 Institut Curie, Paris F-75248, France; 4 INSERM U900, Paris F-75248, France

## Abstract

**Background:**

Predicting which molecules can bind to a given binding site of a protein with known 3D structure is important to decipher the protein function, and useful in drug design. A classical assumption in structural biology is that proteins with similar 3D structures have related molecular functions, and therefore may bind similar ligands. However, proteins that do not display any overall sequence or structure similarity may also bind similar ligands if they contain similar binding sites. Quantitatively assessing the similarity between binding sites may therefore be useful to propose new ligands for a given pocket, based on those known for similar pockets.

**Results:**

We propose a new method to quantify the similarity between binding pockets, and explore its relevance for ligand prediction. We represent each pocket by a cloud of atoms, and assess the similarity between two pockets by aligning their atoms in the 3D space and comparing the resulting configurations with a convolution kernel. Pocket alignment and comparison is possible even when the corresponding proteins share no sequence or overall structure similarities. In order to predict ligands for a given target pocket, we compare it to an ensemble of pockets with known ligands to identify the most similar pockets. We discuss two criteria to evaluate the performance of a binding pocket similarity measure in the context of ligand prediction, namely, area under ROC curve (AUC scores) and classification based scores. We show that the latter is better suited to evaluate the methods with respect to ligand prediction, and demonstrate the relevance of our new binding site similarity compared to existing similarity measures.

**Conclusions:**

This study demonstrates the relevance of the proposed method to identify ligands binding to known binding pockets. We also provide a new benchmark for future work in this field. The new method and the benchmark are available at http://cbio.ensmp.fr/paris/.

## Background

Predicting which molecules can bind to a given binding site of a protein with known 3D structure is important to decipher the protein function, and useful in drug design to identify drug precursors or predict potential side effects if a drug candidate is predicted to bind to many protein pockets. A classical assumption in structural biology is that the 3D structure of a protein is related to its molecular function, i.e., the nature of its partner molecules. Most available programs for structure visualization provide tools for 3D structure superposition and comparison, which may help to predict the nature of a protein ligand from those of other proteins with overall similar 3D structure [1]. However, proteins that do not display any overall sequence or structure similarity may present similar binding sites, and consequently also share similar ligands. Therefore, comparison of binding pockets is a more appropriate approach in order to predict if two proteins bind similar ligands [2], and many ligand prediction methods rely on local 3D comparisons at the binding site, using various ways to perform the comparison. For example, [3] compared pockets described with real spherical harmonic expansion coefficients, [4] used a specialized geometric hashing procedure as the core of the SitesBase web server, [5] developed a method that detects multiple common sets of points. An approach proposed by [6] is based on the representation of binding pockets by triangle-discretized spheres. [7] and [8] considered graph-based representations of binding pockets and applied graph matching algorithms. Finally, [9,10] combines the identification of a binding site on a whole protein 3D structure and its comparison to a reference binding site, using a geometric hashing procedure.

Our contribution in this paper is twofold. First, we propose a new similarity measure to compare binding pockets of proteins. For that purpose, we represent a binding pocket by a cloud of atoms in the 3D space, potentially baring labels such as partial charges or atom types. The method relies on the modeling of local protein structures are rigid bodies, and we therefore represent a protein pocket as a cloud of points with fixed relative positions. The new similarity measure is based on a convolution kernel between clouds of points, and allows to align protein pockets. The method provides a superposition of two pockets even if their corresponding proteins present no overall sequence or 3D structure similarity. One important difference between this approach and most existing methods is that it does not require any pairwise matching of atoms (or superatoms), or residues, in order to compare protein binding pockets. Instead we attempt to capture the similarity of atom densities in the 3D space. This confers smoothness properties to the proposed similarity measure. Second, we propose to use a classification method to predict ligands for target pockets according to their similarity scores with a set of pockets with known ligands. This approach is able to handle the difficult case where different families of pockets binding the same ligand are present. This may be observed when the ligand is flexible and can be bound in various conformations by pockets displaying different topologies.

An important question debated is how to compare the quality of similarity measures. We underline that it is not possible to define an intrinsic quality for a similarity measure, because there is no absolute reference. Similarity measures can only be compared according to the question of interest.

Here, we evaluate quality of similarity measures with respect to their ability to predict a ligand for a pocket. Although the area under ROC curves (AUC scores) are commonly used [2], we show that classification-based scores better compare the performances of similarity measures for ligand prediction.

We test our method on a benchmark proposed by other authors, in order to compare our new method to other published algorithms. We also test the methods on a new benchmark containing non redundant protein pockets binding ligands of similar sizes, typical of that of drug molecules, corresponding to a more realistic problem. We provide this new dataset as a publicly available benchmark.

## Methods

### Convolution kernel between clouds of atoms

In our model, a binding pocket is described by a set of atoms in the 3D space. Our objective is to construct a similarity measure between pockets, which may be used to identify pockets binding the same ligand.

Let  denote a binding pocket consisting of *N *atoms, where *x*_*i *_∈ ℝ^3 ^is a 3D vector representing atom coordinates, and *l*_*i *_is a label (discrete or real valued) that may be used to store additional information on the atoms (for example, atom type, atom partial charge, or amino acid type).

A classical approach for pocket comparison is to iteratively align two pockets and further count the number of overlapping atoms, usually within a tolerance of 1 Å. Different implementations of this principle can be found in such methods as the Tanimoto index [11], the SitesBase algorithm (Poisson index, [12]), or the MultiBind algorithm [5]. The alignment is made to maximize the number of overlapping atoms, which is generally a good indicator of pocket similarity. However, atoms may have different positions but play equivalent roles in ligand binding (for example, the side chain of a basic residue may bind a phosphate group of an ATP molecule from different positions), and the role of one atom in one pocket may be played by a group of atoms in another one. These observations suggest the idea of an alternative smooth score which would not count the number of overlapping atoms, but rather use a weighted number of atoms having similar positions. We first consider the case where labels are ignored, and only atom coordinates are used to measure the similarity between pockets. Then, we explain how the information on atom labels may be introduced in the new similarity measure.

Given two pockets *P*_1 _and *P*_2 _the similarity measure *K*(*P*_1_, *P*_2_) is defined as follows(1)

In fact, this similarity measure defines a positive definite kernel, i.e. it may be considered as a true scalar product on atom clouds that represent binding pockets [13]. Implicitly, it defines the following distance between pockets, which has all standard properties of a true metric (non-negativity, identity of indiscernibles, symmetry, triangular inequality):(2)

The parameter *σ *characterizes the sensitivity of the similarity measure (1) to points relative displacements. When *σ *is small, only atoms which are close to each other significantly contribute to *K*(*P*_1_, *P*_2_). On the contrary, when *σ *is large, almost all pairs of atoms contribute to *K*(*P*_1_, *P*_2_). The kernel (1) is an example of a convolution kernel [14,15] between sets of points. Alternative kernels may be constructed by substituting the Gaussian kernel  by any other kernel between 3D vectors *x*_*i *_and *y*_*j*_

Alternatively, the kernel (1) defined between sets of points can also be thought of as a kernel between mass distribution functions estimated from sets of points [16]. More precisely, let us represent each binding pocket *P*_*i *_by a distribution of masses defined as the sum of Gaussian functions with bandwidth *σ*/ and centered on the pocket atoms, namely:

Then kernel (1) between pockets *P*_1 _and *P*_2 _can be recovered, up to a scaling constant, as the scalar product in *L*_2_(ℝ^3^) between the associated mass distributions:

where *C *is a positive constant. In particular, the distance (2) between pockets can be thought of as the *L*_2_(ℝ^3^) distance between the corresponding mass distributions, namely:

This probabilistic interpretation shows that, intuitively, the similarity score is preserved as far as the corresponding mass distributions are stable. It is therefore robust to small uncertainty in 3D coordinates.

However, formula (1) is not fully appropriate in practice, because the proposed measure is not invariant upon rotations and translations of the binding pockets. Therefore, we define a similarity measure *sup-CK *as the maximum of (1) over all possible rotations and translations of one of the two pockets:(3)

where *R *is an orthonormal rotation matrix and *y*_*t *_is a translation vector. *Sup-CK *is not a positive definite measure anymore, but can still be used as a similarity score. In particular, the interpretation of the similarity as a comparison of mass densities is still valid after the rigid motion. Furthermore, to evaluate *sup-CK*, we now need to maximize a non-concave function over the set of rotations and translations, which may have many local maxima. Exact maximization of this non-concave function is a hard optimization problem. An approximate solution can be estimated by running a gradient ascent algorithm, starting from many different initial points, and taking the best local maximum. Choosing initial points near the global optimal can then help find a better solution and accelerate the optimization. In the case of binding pockets, we found experimentally that, rather than starting from random initial points, a good approximation of the optimal translation vector *y*_*t *_is the vector which translates the geometric center of *P*_2 _into the geometric center of *P*_1_:

Similarly, an approximation of the optimal rotation matrix *R *is the rotation that superposes the first principal axis of *P*_2 _with the first principal axis of *P*_1_, the second one with the second one, and the third one with the third one. Since principal vectors are defined up to a sign, the two signs for all principal vectors of one of the pockets have to be tested (there are 2^3 ^= 8 combinations, each combination defining one initial point). If some of the pocket axes have close lengths, it may also be interesting to consider rotations which superpose the first principal axis of one pocket with the second principal axis of the other one.

Gradient ascent method requires to calculate the gradient of the function in (3) with respect to *R *and *y*_*t*_. Calculation of the gradient components related to *y*_*t *_is straightforward:

Since the set of rotation matrices is a 3D manifold embedded in 9D space, we cannot differentiate (1) with respect to each element of matrix *R*.

Therefore, we use the Euler representation of the rotation matrix:(4)

where *R *is expressed as a function of (*ϕ*, *θ*, *ψ*) ∈ [0;2*π*)^3^. We can now calculate the derivatives of the maximand in (3) with respect to (*ϕ*, *θ*, *ψ*). For instance:

This optimization step defines the best pocket superposition, according to the *sup-CK *similarity measure.

As mentioned above, it may be interesting to use additional information on binding pocket atoms, such as atom types or charges. Let us suppose that this information is represented by labels *l*_*i *_(which may be discrete or real variables, or multidimensional vectors) and that it is associated to a similarity measure. For example, to measure the similarity between categorical labels like atom types, one can use the Dirac function . In our experiments, we used atom partial charges as atom labels, with a Gaussian kernel . Of course, other similarity measures may be employed.

These atom labels can be used to re-weight the contribution of two atoms *x*_*i *_and *y*_*j *_by *K*_L_(*l*_*i*_, *l*_*j*_) in (3):(5)

where parameter *λ *controls the sensitivity of our measure to atom labels, for example to partial charges. When *λ *is large, the impact of labels is negligible, which corresponds to a purely geometrical approach. When *λ *is close to zero, only pairs of atoms which have the same partial charge contribute to our measure. In general, the smaller *λ*, the greater the contribution of the atom labels to the binding pocket similarity measure. Since the function *K*_*L *_does not depend on *R *and *y*_*t *_in (5), the same optimization procedure for pockets superposition can be used to optimize (3) or (5). Finally, it is important to notice that the *sup-CK *measure of similarity can be used to compare *any *set of atoms in 3D. As mentioned in the introduction section, the superposition method and the similarity measure may be applied to superpose and compare pockets, even when they belong to proteins displaying no sequence and no overall structure similarity. This point will be illustrated in Results on the example of two unrelated ATP binding proteins.

### Related methods

In the following, we briefly recall the principals of a few other methods proposed to measure similarity between pockets, because we compare them to the *sup-CK *method defined in the present study.

#### Spherical harmonic decomposition (SHD)

[3] proposed to model pockets by star-shapes built using the SURFNET program. The star-shape representation is defined by a function *f *(*θ*, *ϕ*), representing the distance from the pocket center to the pocket surface for a given (*θ*, *ϕ*),. To measure the similarity of binding pockets *P*_1 _and *P*_2_, the corresponding functions *f*_1 _and *f*_2 _are first decomposed into spherical harmonics, and the pocket similarity is then computed as the standard Euclidean metric between vectors of decomposition coefficients.

[2] presented three different variants of *SHD*, using only the shapes or the sizes of the binding pockets (keeping only the zero-th order in the spherical harmonics expansion), and their combination. In the Results section, we recall the results that they obtained with the combination, because it provided the best performance.

#### Poisson index (sup-PI)

As mentioned in the Introduction, many binding pockets similarity measures are based on pocket alignment with further counting of overlapping atoms. This kind of approach is used in the *Poisson index *model [12]. More precisely, the *Poisson index *model is based on a normalized number of overlapping atoms , where *L *is the number of overlapping atoms, and #*P*_1 _and #*P*_2 _are the numbers of atoms in *P*_1 _and *P*_2_, respectively. The *PI *score may be computed for any pocket superposition method. While [12] used the geometric hashing algorithm, we used the superposition made by the *sup-CK *method, with further superposition refining to maximize the number of overlapping atoms.

#### Multibind

[5] represents pockets by pseudo-atoms labeled with physico-chemical properties. Pockets are aligned using a geometric hashing technique. This algorithm was mainly designed for multiple alignment of binding sites, but it may be used for pairwise alignment of pockets, as performed in this study.

#### Other simple methods

We also consider two simple methods based on the comparison of simple binding pockets characteristics. These methods represent each pocket by an ellipsoid constructed on the basis of the pocket's principal axis. The first one, referred to as *Vol*, estimates the similarity between pockets *P*_1 _and *P*_2 _by the absolute value of the difference between the volumes of their corresponding ellipsoids:

*Vol*(*P*_1_, *P*_2_) = |*Vol*(*P*_1_) - *Vol*(*P*_2_)|. The second one, called *Princ-Axis*, estimates the similarity score between pockets by , where  and  are the lengths of the three principle axis of pockets *P*_1 _and *P*_2_, respectively.

#### Combination of sup-CK and Vol

Since volume information was found to be important by [2], we also tested a linear combination of the *sup-CK *and *Vol *methods, called *sup-CK-Vol*, where the coefficient of linear combination is learned as other model parameters (*σ*, *λ*, or the distance cutoff *R *discussed in the Datasets section) in the double cross validation scheme. This linear combination takes advantage of the *Vol *method to separate pockets binding ligands of very different sizes like PO4 and NAD, and of the *sup-CK *algorithm to allow finer discrimination.

#### Sequence

To compare our method based on local 3D similarity to a simple and classical approach based on sequence comparison, we conducted a pairwise alignment of all protein sequences for the different datasets, in order to build a matrix of distance between proteins. This matrix was built with the algorithm of Needleman and Wunsch, using the default settings [17,18].

### Performance criteria

There are various ways to measure the similarity between binding pockets, and some of them were discussed in the previous section. To evaluate the quality of a given similarity measure, one may compare it to some "ideal" similarity measure, but the problem is that such measure does not exist. As an example, if two alternative measures SM1 and SM2 compare two pockets P1 and P2 so that SM1(P1, P2) = 0.3 and SM2(P1, P2) = 0.4, there is no way to decide which one is the best, because we do not have any absolute reference. The choice of the optimal measure, thus, depends on the problem of interest. In the context of ligand prediction, the quality of a similarity measure can be evaluated according to its ability to cluster together pockets that bind the same ligand. This can then help to predict ligands for previously unseen pockets. To evaluate this clustering ability, we considered two different scores.

#### AUC score

**Figure 1:**
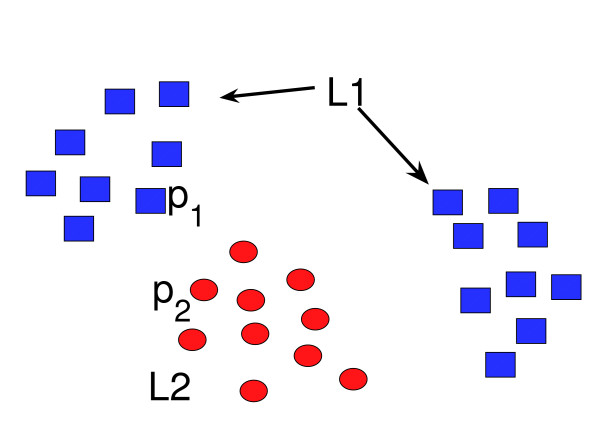
**AUC score versus classification error as an evaluation of binding pocket similarity measure**. Red circles represents pockets fixing ligand *L*_1_, blue squares represents pockets fixing ligand *L*_2_. The AUC score does not reflect the fact of good pocket clusterization, while the classification error does.

[2] used the AUC score which is computed as follows. Let us consider a set of pockets (*P*_1_,.., *P*_*N*_) and a similarity measure *SM*. To estimate the AUC score of a given pocket P_*_, we rank all other pockets according to their similarity to P_*_, *SM*(*P*_*i*_, P_*_) (descending order), and we plot the ROC curve, i.e., the number of pockets binding the same ligand versus the number of pockets binding a different ligand among the top *n *pockets, when *n *varies from 0 to *N*. The quality of *SM *is measured by the surface of the area under the ROC curve, which defines the AUC score. An "ideal" *SM *function will rank all pockets binding the same ligand as P_* _on the top of the list, leading to an AUC score equal to 1.0. On the contrary, if these pockets have random positions in the ranked list, the AUC score will be equal to 0.5 (worst possible case). Finally, the overall AUC score of a method equals its mean value over all pockets. While the AUC score represents an intuitive and classical way to evaluate the quality of similarity measures, it may fail in some situations. Consider the case of a dataset containing two types of pockets *L*_1 _and *L*_2 _(i.e. binding two different ligands), and a similarity measure that correctly clusters pockets according to their type. If clusters are close to each other (see clusters A and C in Figure 1), the AUC score of pockets situated near the border (pockets *p*_1 _and *p*_2 _in Figure 1) will below. The situation becomes even worse if pockets binding ligand *L*_1 _form several clusters, as shown in Figure 1, leading to low AUC scores for almost all pockets binding ligand *L*_1 _This similarity measure will have an overall poor AUC score on this dataset, although it produces perfect separation of pocket types. This may happen when the database contains proteins that underwent convergent evolution, or that bind the same ligand under very different conformations. Therefore, a poor AUC score does not necessarily correspond to a poor pocket separation, and AUC scores may not be suited to evaluate the quality of similarity measures with respect to the question of ligand prediction.

#### Classification error

These remarks lead us to employ another quality score based on a classification error. To estimate the quality of the similarity measure *SM*, we try to predict a ligand (class) for each pocket from that of its neighbors. The smaller the classification error (proportion of bad predictions), the better the similarity measure. In this work, we used a K nearest neighbors (KNN) classifier. To evaluate the classification error, we applied a leave-one-out double cross validation methodology. Namely, each pocket P from the dataset is considered one by one, and all other pockets are used as the training set. Parameters of the model (*K *-- number of neighbors, *σ *and *λ *in the case of the *sup-CK *method) are estimated on the training set via cross-validation technique, and the class (i.e. the ligand) of the pocket P under consideration is predicted using the training set and the estimated parameters of the model. More precisely, in the case of a dataset containing 100 proteins, double cross validation is performed according to the following scheme: each of the 100 pockets is extracted in turn from the dataset in a leave one out procedure. Then, each of the other 99 pockets is selected in turn and its class is predicted from the 98 remaining pockets. This operation is repeated for different values of *σ *and *λ*, and the σ* and λ* values providing the highest number (over 99) of well predicted pockets are retained and used to predict a class for the initially extracted pocket. Note, that all datasets contained proteins that presented less than 30% global sequence identity [17], to ensure that there were no duplicates or very close elements in the datasets. This allowed to use a leave-one-out scheme without risk of bias.

### Data

For all protein structures, binding pockets were extracted as follows: protein atoms situated at less than *R *Å of one of the ligand atoms were selected, where *R *is a parameter of the model (as the number of neighbors *k*, or the *σ *and *λ *parameters), and is also learned in the double cross-validation scheme. In most cases, the optimal value of *R *was found to equal to 5.3 Å, a value which was retained in this study. However, experiments where *R *is varied are also presented in the discussion section. Finally, pockets are represented by 3D clouds of atoms labeled by their partial charge, attributed according to the GROMACS (FFG43a1) force field [19]. Atom partial charges were assigned according to the protein structure alone, in absence of the ligand. However, the presence of a ligand would potentially modify these calculated charges, but this could not be taken into account since the method aimed at predicting the ligand. Other labels representing chemical properties such as amino-acid type, hydrogen donor or acceptor, or hydrophobic atom could be included.

We considered three benchmark datasets. The first one, proposed by [2] and referred to as the *Kahraman dataset*, comprises 100 protein crystal structures in complex with one of ten ligands (AMP, ATP, PO4, GLC, FAD, HEM, FMN, EST, AND, NAD). The second one is an extended version of the Kahraman dataset (called *extended Kahraman Dataset *below), in which we added protein structures in complex with one of the same ten ligands, leading to a total of 972 crystal structures (see Additional file 1). The added proteins presented pairwise sequence identities less or equal to 30%, to avoid potential bias by inclusion of close homologues.

The Kaharaman dataset comprises ligands of very different sizes and chemical natures, as shown in Table 1. However, the real challenge is to test methods on pockets that bind ligands of similar sizes. Therefore, we created a third dataset comprising 100 structures of proteins in complex with ten ligands of similar size (ten pockets per ligand), see Table 2. This dataset will be referred to as the *Homogeneous Dataset *(HD) (see Additional file 2). The results presented below on this dataset may constitute a new benchmark for future work in the same area.

**Table 1:** Ligands descriptors for the Kahraman dataset

ligand	atoms count	Molecular weight	hydrogen-bond acceptors	hydrogen-bond donors	Rotatable bonds
AMP	23	345.21	9	4	4
ATP	31	503.15	13	4	8
PO4	5	95.98	3	1	0
GLC	12	180.16	6	5	1
FAD	53	785.55	15	10	13
HEM	43	616.49	4	2	8
FMN	31	456.34	8	6	7
EST	20	272.38	1	2	0
AND	21	288.42	2	1	0
NAD	44	663.43	14	9	11

**Average**	**28.3 ± 15.0**	**420.7 ± 222.8**	**7.5 ± 5.1**	**4.4 ± 3.2**	**5.2 ± 4.9**

AMP: adenosine monophosphate, ATP: adenosine-5'-triphosphate FAD, flavin-adenine dinucleotide, FMN: flavin mononucleotide, GLC: alpha-D-glucose, HEM: protoporphyrin containing Fe, NAD: nicotinamide-adenine-dinucleotide, PO4: phosphate ion, AND: 3-beta-hydroxy-5-androsten-17-one, EST: estradiol.

**Table 2:** Ligands descriptors for the homogeneous dataset

ligand	Atom count	Molecular weight	hydrogen-bond acceptors	hydrogen-bond donors	Rotatable bonds
PMP	16	247.17	4	4	4
SUC	23	342.3	11	8	5
LLP	24	361.33	5	6	11
LDA	16	229.4	1	0	11
BOG	20	292.37	6	4	9
PLM	18	255.42	2	0	14
SAM	27	399.45	8	7	7
U5P	21	322.17	8	3	4
GSH	20	306.32	6	6	11
1PE	14	208.25	5	1	11

**Average**	**19.9 ± 4.0**	**296.4 ± 61.5**	**5.6 ± 3.0**	**3.9 ± 2.9**	**8.7 ± 3.5**

PMP: 4'-deoxy-4'-aminopyridoxal-5'-phosphate, SUC: sucrose, LLP: 2-lysine(3-hydroxy-2-methyl-5-phosphonooxymethyl- pyridin-4-ylmethane), LDA: lauryl dimethylamine-N-oxide, BOG: b-octylglucoside, PLM: palmitic acid, SAM: S-adenosylmethionine, U5P: uridine-5'-monophosphate, GSH: glutathione, 1PE: pentaethylene glycol.

## Results

All methods were tested on three datasets described in the Data section. The performance of all methods is evaluated on the basis of the AUC score and of the classification error (see Performance criteria). The *sup-CK *method is compared to *sup-PI*, *SHD*, *Vol*, *Princ-Axis *and *MultiBind *algorithms (see Related methods). Among the pocket extraction methods used in the *SHD *approach, we considered the results corresponding to the Interact Cleft Model, which is similar to our pocket extraction method, and allows to compare the *sup-CK *and *SHD *approaches. Algorithms, benchmark datasets and distance matrices for the SupCK method are available at http://cbio.ensmp.fr/paris/.

### Kahraman Dataset

Results of all methods on the Kahraman Dataset are presented in Table 3. According to the AUC score, all methods improve the baseline value of 0.5 corresponding to a random ranking, and simple methods like *Vol *and *Princ-Axis *give surprisingly good results, for example, there is no significant difference between the AUC score of *Vol *and the AUC score of the best performing method *sup-CK*_*L*_*-Vol*. The same effect was observed by [2] when they used simple measures based on comparison of pockets sizes.

**Table 3:** Performance on the Kahraman benchmark

Method	AUC	CE
sup-CK	0.858 ± 0.14	0.36
sup-CK_*L*_	0.861 ± 0.13	0.27
sup-CK-Vol	0.889 ± 0.14	0.34
sup-CK_*L*_-Vol	0.895 ± 0.12	0.26
Vol	0.875 ± 0.14	0.39
Princ-Axis	0.853 ± 0.13	0.35
sup-PI	0.815 ± 0.13	0.42
SHD	0.770	0.39
MultiBind	0.715 ± 0.17	0.42

Sequence	0.55 ± 0.08	0.8

Performance for each algorithm is evaluated by its mean AUC score and by its classification error (CE), averaged over all pockets. (AUC score for SHD are taken directly from [2], CE scores are estimated from data provided by authors).

**Figure 2:**
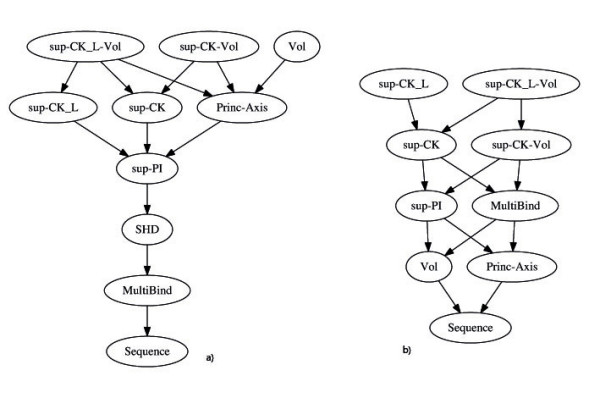
**Relationship between AUC performances of the methods tested**. (a) on the Kahraman dataset (b) on the Homogenous dataset. Each node corresponds to a particular method, parent nodes perform significantly better than child nodes according to the Wilcoxon signed-rank test.

As expected, the score obtained using the sequence alignment is close to the baseline value, indicating that this approach is not suitable to the problem of predicting ligand when sequences are very different. The AUC scores of *sup-CK-Vol *(with or without partial charges) are better than those of all other methods, except for *Vol*, according to the Wilcoxon signed-rank test (see Figure 2a). The best results are obtained by the *sup-CK-Vol *algorithm, which seems to benefit from the association of volume information and of more subtle geometric details provided by the *sup-CK *algorithm. Another observation, is that information on atom partial charges does not significantly improve the AUC score of the *sup-CK *methods.

To evaluate the classification error, we tried to predict a ligand (a class) for each pocket using the k-nearest neighbors classifier (see Performance criteria). Note that in a ten class (10 ligands) classification problem, a random classifier would have an error of 0.9, which represents baseline performance for all classifiers.

Table 3 shows that methods with higher AUC scores tend to have smaller classification errors, but this correlation is not strict. For example, the *SHD *and *Vol *methods have the same classification error, although the latter displayed a better AUC score than the former. Conversely, the *sup-CK *and *sup-CK*_*L*_-*Vol *methods had similar AUC scores, but the latter performs much better than the former in terms of classification error. This indicates that the AUC score is not appropriate to compare the quality of similarity measures with respect to the problem of ligand identification, and underlines the interest of the classification approach.

The *sup-CK *and *sup-CK-Vol *algorithms have lower classification errors than other methods, which means that they are well suited to the problem of ligand prediction. Interestingly, atom partial charges information significantly reduces classification errors of both methods, which was not the case for AUC scores. The use of additional atom labels such as amino-acid type, hydrogen donor or acceptor, or hydrophobic atom may again improve the quality of ligand prediction.

No method reaches the AUC score of 1.0, or perfectly predicts the ligands. Several remarks might explain this fact. First, pockets have to be extracted from the protein structure. Whatever the employed method might be, it is difficult to extract all atoms interacting with the ligand, and only these atoms. In particular, atoms that do not interact with the ligand might have been included in the pockets, which could reduce the observed similarity between pockets that bind this ligand. Second, ligands are flexible molecules that can adopt different conformations. Therefore, protein pockets that bind the same ligand may display various shapes. In such situations, correct prediction is still possible if the learning dataset contains pockets in which the ligand conformations correctly samples its accessible conformational space. The present dataset contains only 10 pockets per ligand, which might be too small for the most flexible ligands.

When analyzing results in Table 3, one must remember that the *Vol *and *Princ-Axis *methods do not require pockets superposition, while all other methods do. The superposition algorithms of the latter are different, which contributes to the observed scores. However, the *sup-PI *and *sup-CK *methods only differ by their similarity measures. After superposition, *sup-PI *requires to determine the number of overlapping atoms, while *sup-CK *relies on a weighted number of atoms having close positions. This seems to confer some smoothness properties to the latter, and robustness with respect to variations observed between pockets binding the same ligand.

**Figure 3:**
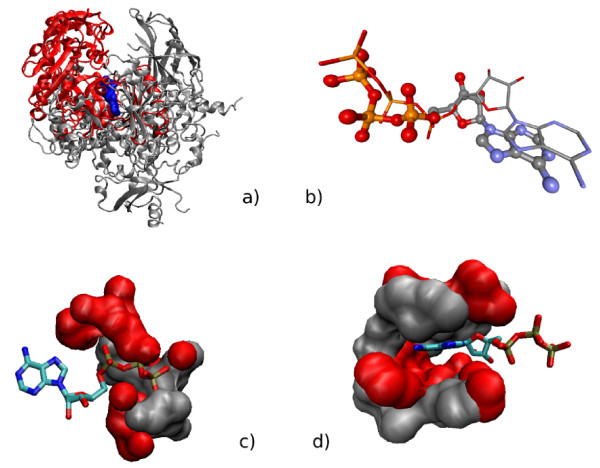
**Superposition of the binding pockets of two structurally different proteins binding ATP**. A) overall structures of pdb: PDB: 1E8X in grey and PDB: 1DV2 in red superposed according to their binding sites using Sup-CK. ATP molecules are represented in blue. B) Superposition of the ATP molecules from PDB: 1DV2 and PDB: 1E8X when their binding sites are superposed. C) Positively charged protein regions around ATP molecules of PDB: 1E8X in grey and PDB: 1DV2 in red. D) Protein hydrophobic patches around ATP molecules of PDB: 1E8X in grey and PDB: 1DV2 in red.

An important point mentioned in Background is that pocket superposition with *sup-CK *does not require any sequence or structure similarities between the corresponding proteins. To illustrate this property, we analyzed in more details the results for ATP-binding proteins of this dataset. For example, the biotin carboxylase from *E. coli *(452 residues in PDB: 1DV2), and the phosphoinositide 3-kinase (961 residues in PDB: 1E8X) are unrelated proteins. They present no sequence similarity (they cannot be aligned), and their overall structures are totally different, as shown in Figure 3a. However, they bind ATP in similar conformations. When these two pockets are aligned with the *sup-CK *algorithm, their corresponding ATP molecules are found correctly superposed, as shown in Figure 3b, although the *sup-CK *algorithm only uses protein atoms.

Moreover, similar residues, playing equivalent roles in ATP binding, are found in equivalent positions in the superposed structures. In particular, N951 and K807 interact with the *γ *phosphate of ATP in PDB: 1E8X and are found close respectively to K288 and H236 that play the same role in PDB: 1DV2. We also observe that, K833 interacting with the *β *and *α *phosphates of ATP in PDB: 1E8X, is found close to K116 in PDB: 1DV2 after pockets superposition. These residues form equivalent positively charged regions, as shown in Figure 3c. Similarly, the hydrophobic region interacting with the adenine ring of ATP in PDB: 1E8X and involving residues W812, I831, I879, I881, V882, A885, M953, F961, and I963 is equivalent to the hydrophobic region involving residues V131, V156, I157, L204, L278, I287, I437 in the superposed PDB: 1DV2 structure. These hydrophobic patches overlap after pockets superposition, as shown in Figure 3d. Overall, these observations indicate that the *sup-CK *algorithm proposed a reasonable superposition for these two unrelated ATP-binding pockets.

**Figure 4:**
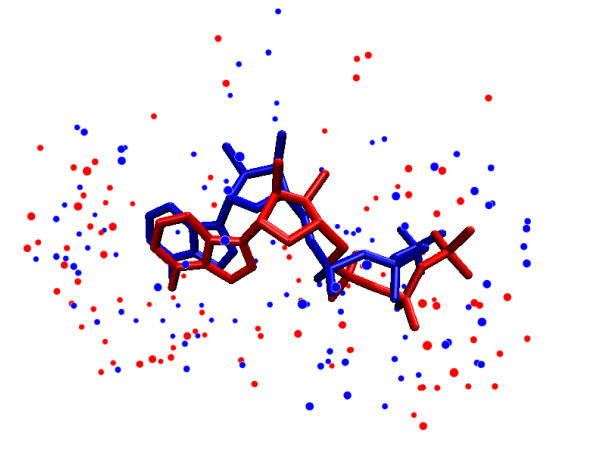
**Alignment two ATP binding pockets**. Alignment of two ATP pockets made by *sup-CK*, atoms of each pockets are represented by blue and red points, two ATP ligands are traced in licorice.

Figure 4 shows the alignment of the two pockets, extracted from PDB: 1E8X and PDB: 1DV2 as clouds of atoms, and superposed by *sup-CK*. Note, that *sup-CK *did not try to superpose individual atoms, but rather superposes atom sets.

### Extension of Kahraman dataset

To evaluate the ability of the *sup-CK *method to improve its performance when trained on a larger dataset, we considered an extension of Kahraman dataset consisting of 972 of non redundant pockets that bind one of the 10 ligands of the original dataset (see Data). Therefore, the new dataset consists of 100 Kahraman pockets and 872 new pockets from the PDB.

Table 4 presents the classification errors observed on this dataset for different algorithms. Note that in the case of the *sup-CK *methods, the parameters were optimized on the original Kahraman dataset of 100 proteins. Column A presents the classification errors when all 972 pockets are used in the leave-one-out procedure. It shows that all methods improve when the dataset is larger.

**Table 4:** Classification error on the extended Kahraman benchmark

Method	A	B	C
sup-CK_*L*_	0.19	0.21	0.18
sup-CK_*L*_-Vol	0.18	0.19	0.18
Vol	0.32	0.39	0.31
Princ-Axis	0.22	0.27	0.21
sup-PI	0.24	0.33	0.23

Classification error for all algorithms on the extended Kahraman dataset. Column A - classification error evaluated on all 972 pockets. Column B Proportion of wrong predictions among the original 100 Kahraman pockets extracted from column A, i.e. classification error evaluated on 100 Kahraman pockets when all 972 pockets are used in the leave-one-out procedure. Column C - classification error evaluated on the 872 new pockets, when the 100 Kahraman pockets are not used in the leave-one-out procedure.

However, *sup-CK*_*L *_provides the best performance. The quality of its predictions might again improve by including more structures available at the PDB. Column B presents the results on the 100 original pockets extracted from those presented in column A. It shows that 79% of the binding pockets of the original Kahraman dataset were correctly classified by *sup-CK*_*L*_, compared to 73% when they were classified using only the original dataset (a classification error of 0.27 in Table 3). Finally, column C shows the prediction errors for the 872 new pockets when the 100 original pockets are not used in the leave one out procedure. The scores in this column may be seen as a test on an external independent dataset (as mentioned above, the optimal parameters *σ *and *λ *used here were those learned only on the 100 original pockets). It shows that the performance of the *sup-CK *methods does not degrade on the 872 new pockets, and remains above those of the other methods.

**Figure 5:**
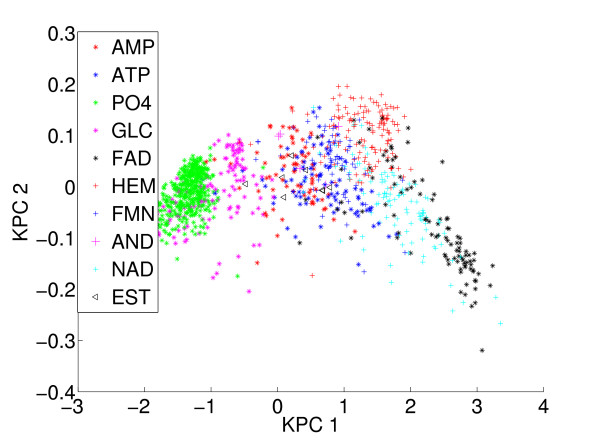
**Projection of the ext-KD dataset on the first two kernel principal components defined by the similarity measure *sup-CK***. Clustering of binding pockets according to their ligands, which illustrates the performance of this method for ligand prediction.

It is also interesting to study the structure of the dataset according to the metric associated to the *sup-CK *method. We performed kernel principal component analysis [20] on the pockets similarity matrix of the *sup-CK *method (this matrix is not positive definite, but we can extract principal components associated to the largest positive eigenvalues). Figure 5 represents the projection of 972 binding pockets on the first two principal components. Overall, we observe a clustering of binding pockets according to their ligands, which illustrates the good performance of this method for ligand prediction. Looking into more details, we notice that the clusters of pockets that bind ATP, AMP or PO4 overlap. Indeed, proteins that bind ATP usually also bind AMP or PO4, although with different affinities. Furthermore, some pockets (for example pockets that bind GLC or FAD) are found far from their main cluster, or form secondary clusters, which illustrates that pockets having different geometrical characteristics may bind the same ligand. In the classification approach employed here, prediction of a ligand for a given pocket uses the classes of its neighbors, which allows to better predict ligands for pockets belonging to such secondary clusters.

### Homogeneous dataset (HD)

The Kahraman dataset contains ligands of very different sizes, which might not be typical of real problems. Therefore, we built the Homogeneous dataset because it was important to test methods on a benchmark containing pockets binding ligands of more similar sizes.

Table 5 shows that the performances of all algorithms are lower than on the Kahraman dataset, which illustrates that the Homogeneous dataset is a more difficult benchmark. *Vol *and *Princ-Axis *display stronger degradation of performances, with AUC scores of 0.65, and classification errors of 89% and 71%, respectively. The latter must be compared to the baseline value of 90% error for a random classifier for ten classes (ten ligands). This illustrates that size information is less discriminative on this dataset, as expected. All other methods display a stronger improvement with respect to the baseline. Interestingly, although the AUC scores of the simple *Vol *and *Princ-Axis *methods are only 5 to 10% lower than those of all other methods, their classification error is much worse, and *Vol *does not behave better than a random classifier. This again underlines the interest of the classification error score to compare the performances of similarity measures for ligand prediction.

**Table 5:** Performance on the HD benchmark

Method	AUC	CE
sup-CK	0.710 ± 0.19	0.47
sup-CK_*L*_	0.752 ± 0.16	0.38
sup-CK-Vol	0.722 ± 0.18	0.46
sup-CK_*L*_-Vol	0.766 ± 0.17	0.38
Vol	0.648 ± 0.15	0.89
Princ-Axis	0.650 ± 0.18	0.71
sup-PI	0.702 ± 0.19	0.47
MultiBind	0.69 ± 0.14	0.48

Sequence	0.577 ± 0.09	0.83

Performance for each algorithm is evaluated by its mean AUC score and by its classification error (CE), averaged over all pockets.

The best AUC score is obtained by the *sup-CK*_*L*_-*Vol *algorithm. The AUC scores of all other methods are significantly lower according to the Wilcoxon signed-rank test (see Figure 2b), except *sup-CK*_*L*_. Indeed, volume information only provides a slight improvement of 1%, compared to 3% on the Kahraman dataset. On the contrary, information on partial charges leads to an improvement of 4% for the *sup-CK *and *sup-CK-Vol *algorithms, which was not observed on the Kahraman dataset. This shows that addition of physico-chemical information is critical to better compare pockets of similar sizes. The lowest classification errors are obtained by the *sup-CK*_*L *_and *sup-CK*_*L*_*-Vol *algorithms, which again shows that volume information is not critical on this benchmark. On the contrary, partial charge information leads to an improvement of 9% between *sup-CK *and *sup-CK*_*L*_, and of 8% between *sup-CK-Vol *and *sup-CK*_*L*_*-Vol*.

## Discussion

### Computer vision methods

An important topic is the relation between methods for binding pockets comparison and algorithms in field of computer vision for comparison of 3D shapes. A complete review of 3D shapes comparison methods is out of scope of this article, and interested readers may consult [21] for a detailed review. Interestingly, most of the existing methods for binding pocket comparison have an analogue in the domain of computer vision. For example, methods based on real spherical harmonic expansion used in [3] for binding pocket comparison are also discussed by [22,23] in the context of general 3D shape matching. Principles used in another popular method for matching and comparison of 3D forms, called Iterative Closest Point algorithm [24], and its variants are used in *Poisson index *and *MultiBind *algorithms. Examples of approaches based on graph representation of 3D forms and graph matching methods may be found in [7] for binding pockets comparison, as well as in [25] for 3D shapes comparison. Nevertheless, binding pockets are not continuous shapes but discrete clouds of points. They can be transformed into 3D shapes [2,3], but this transformation may be a source of noise. Moreover, a similarity measure between binding pockets should be rotationally and translationally invariant, which is not always the case in computer vision methods. However, we believe that the adaptation of appropriate methods may be very fruitful for the recognition of binding pockets.

### Choice of optimal parameters

**Figure 6:**
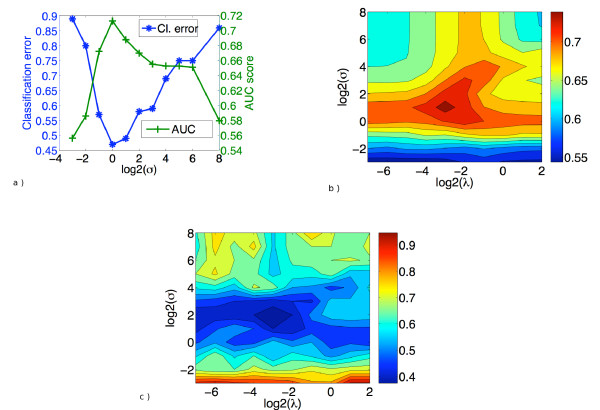
**Performance on the HD dataset**. (a) Mean AUC score and prediction error as functions of *σ *in the *sup-CK *method (pure geometrical version, *λ *= ∞), (b) mean AUC score and (c) classification error as functions of *σ *and *λ *when information on atoms partial charges is used.

An important characteristic of the *sup-CK *algorithm is its ability to adapt to the variability potentially observed between pockets binding the same ligand. The *sup-CK *algorithm presents two parameters, *σ *and *λ*. Parameter *σ *controls the sensitivity of the similarity measure to atoms relative displacements. The larger the variability of pockets binding the same ligand, the greater the value of *σ *should be. Figure 6a shows how the mean (over all pockets) AUC score and classification error vary with *σ *on the Homogeneous dataset. In both cases, the optimum is reached when *σ *is equal to 1. Note that we did not use the same value of *σ *estimated from all pockets. For each pocket, the optimal value was estimated on the basis of the remaining 99 pockets used for training, in a double cross validation scheme, to avoid overfitting to the data. However, we observed that, in most cases (90%), *σ *= 1 was chosen. When information on atom partial charges is used, parameter *λ *(5) conditions the sensitivity of the method to atoms charges. Figures 6b and 6c present the mean AUC score and the classification error as functions of *σ *and *λ*. We observe that for the AUC score, the optimum is reached when *σ *equals 2 and *λ *equals 0.25, while for the classification error the optimal value of *σ *is equal to 4.

**Figure 7:**
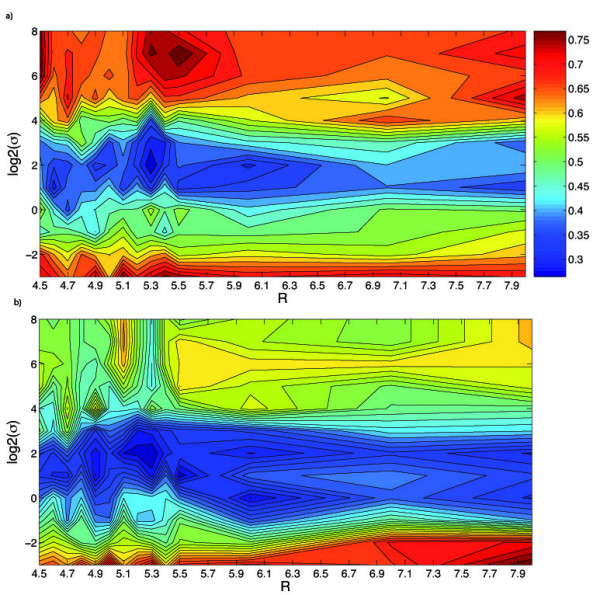
**Classification error of the *sup-CK*_*L *_algorithm**. as a function of *R *and *σ *(*λ *= 0.25): (a) Kahraman dataset, (b) HD dataset.

While in general we suggest to learn these two parameters of the *sup-CK *algorithm on the dataset of interest, we observed that some default values provide good performance in many cases, and that they could be used in dry-runs on new datasets. For example, a good default value for *σ *is 1. This value was optimal for the HD dataset when we used the pure geometrical approach, and it also gave good results on the Kahraman and extended Kahraman datasets. When partial charges are used, i.e. with the *sup-CK*_*L *_algorithm, larger default values for *σ *are recommended (between 2 and 4), and a good default value for *λ *is around 0.25. The radius *R *of the extracted pocket is a parameter of the extraction pocket procedure. Figures 7a and 7b present the classification errors of *sup-CK *as a function of *σ *and *R*, respectively for the Kahraman and the HD datasets. We observe that in both cases, the optimal value of *R *is around 5.3 Å, which corresponds to a good default value. However, Figures 7a and 7b show that the performance of the method is still interesting for values varying between 4.5 and 8 Å. Importantly, they also show that the optimal value of *σ *does not depend on R. Finally, K is a parameter of the K nearest neighbors classifier (KNN classifier). Ideally, it should also be learned, but values of K between 3 and 5 usually work well.

### Robustness of the method with respect to pockets definition

It is important to discuss the impact of using the *R *parameter, a cutoff distance used for pocket definition. This could lead to situations where an atom is excluded from the pocket in one protein, when a similar atom is included in the pocket of another protein to which it is compared. However, as briefly mentioned in the background section, the principle of the method is to compare pockets based on the optimal superposition of their clouds of atoms. The method does not define or use pairwise matching of atoms of the two pockets, as most other available methods do. Figure 4 illustrates this point: the method did not lead to local pairwise superposition of blue and red points, but rather proposed a global superposition of the red and blue atoms densities. Therefore, the method is expected to be robust with respect to potential inclusion or exclusion of a small number of atoms in one of the pockets. As mentioned in the above paragraph, the fact that the performance of the method remains interesting when *R *varies between 4.5 and 8 Å is also an indirect illustration of this idea. One could wonder if the use of atom labels such as partial charges would decrease the robustness of the method with respect to pockets definition using *R*. Indeed, a cutoff distance could split a strong dipole in one of the proteins, and not in the other (for example an N-H group). However, the addition of atom labels like partial charges is only one option of the method. Results using only atom positions (corresponding to a pure geometrical approach) already show good performances. Addition of partial charges labels still improves the results, despite the risk that strong dipoles might have been cut. This can probably be explained by the facts that such events are rare, and that the method searches an overall best superposition of atoms densities, despite possible local mismatches in atoms positions or labels. Nevertheless, it would be interesting to explore other cutoff criteria taking atom labels into account (including other types of labels such as hydrogen bond acceptor, donor,...), in future developments of the method.

### Pocket extraction

We did not tackle the problem of pocket detection, which relies on totally different algorithms than those discussed in this paper, and which was out of the scope of the present study. However, the similarity measured between two pockets strongly depends on pocket definition. We extracted pockets as the set of all protein atoms within about 6 Å of the bound ligand. Similar approaches were used by [2] (Interacted Cleft Model), and similar pockets may also be retrieved by methods like *Q-SiteFinder *[26] without any information on ligand coordinates. Another alternative could be to employ one the various programs that have been developed to locate depressions on protein surfaces, particularly in the case where no holo structure is available [27], or in the case of orphan proteins for which the ligand and the binding site is unknown. However, existing pocket extraction algorithms have difficulty to define the rim of a binding pocket, and tend to extract protein cavities that are larger than the binding pocket itself, as defined by the ensemble of residues involved in ligand binding. Although we observed that our method had some robustness with respect to the definition of the binding pocket, global similarity measures like those proposed in this paper may loose some performance on automatically extracted pockets.

### Protein functions

**Figure 8:**
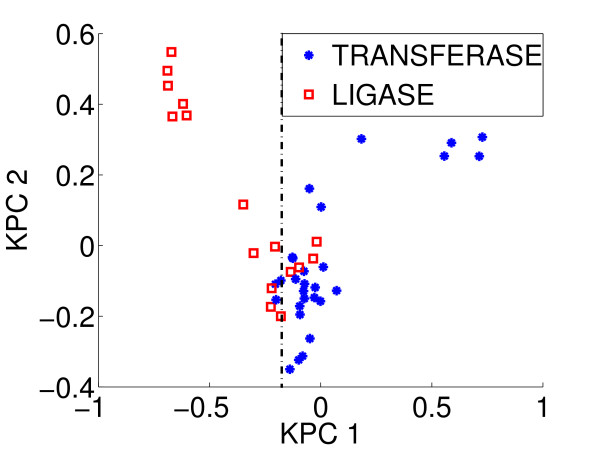
**Projection of ATP binding pockets on the two first kernel principal components of *sup-CK***. Repartition of the ATP binding pockets generated by the *sup-CK *similarity measure on the extended Kahraman dataset. Red squares represent ligases, blue stars represent transferases.

The problem of ligand prediction for proteins is related to the problem of predicting the protein molecular function. We analyzed the repartition of the ATP binding pockets generated by the *sup-CK *similarity measure on the extended Kahraman dataset. Figure 8 presents the projection of ATP pockets annotated as transferases or ligases, on the first two principal components of the similarity matrix associated to *sup-CK*. We observed that these two families of enzymes are essentially separated. Although these are very preliminary results, they show that *sup-CK *method may be a useful tool, in conjunction with other approaches, for the prediction of protein molecular functions.

In the Result Section, we showed the example of the PDB: 1E8X and PDB: 1DV2 unrelated structures, binding ATP in similar conformations, and whose pockets were correctly superposed by the *sup-CK *method. In the case of even more dissimilar pockets, binding ATP in different conformations, *sup-CK *still allows superposition of the pockets so that similar regions overlap. For example, rabbit muscle pyruvate kinase (530 residues in PDB: 1A49) and *E. coli *7,8-dihydro 6-hydroxymethylpterin pyrophosphokinase (158 residues in PDB: 1DY3) of the Kahraman dataset have no sequence or structures homologies, and bind ATP in different conformations as shown in Figure 3a. However, according to the *sup-CK *superposition of these two pockets, shown in Figure 3c, the two patches of hydrophobic residues that interact with the adenine ring of ATP are found to overlap. Note that these two pockets where correctly classified by *sup-CK *(an ATP ligand was correctly predicted), on the basis of other more similar pockets present in the dataset.

Nevertherless, a reasonable pocket superposition for these highly different proteins with significant pockets deviations, was proposed by the *sup-CK *method.

### Apo structures

The *sup-CK *algorithm had a good performance in ligand prediction for holo structures. It also showed robustness with respect to atom displacements. This is an important characteristic for future application of the method to real case studies where the ligand is unknown, and one must extract pockets from apo structures. Local structural rearrangements are frequent upon ligand binding, and methods displaying some smoothness with respect to atoms positions are required when working with apo structures. This would also be necessary for proteins with no available experimental structure but for which a homology model can be constructed, since the modeled pocket may somewhat differ from the true, but unknown, pocket. We expected that, for large flexible ligands, the performance of the *sup-CK *method might decrease, but this was not observed for the two datasets that we used (Kahraman dataset and Homogeneous dataset). However, we cannot rule out the possibility that this could be observed if the method is trained on other small training datasets.

### Computational issues

The running time of the *sup-CK *method depends on the value of the stopping criterion used in the gradient ascent method, and on the number of atoms. In our experiments, the algorithm running time varied between 0.2 and 1.3 seconds (2.5 GHz CPU) per pockets pair. This running time is already quite reasonable to process large protein databanks. The method is presented on datasets of moderate sizes because our aim was to validate the methodology. However, it can be applied on ligand prediction problems, where the number of pockets (and ligands) included in the learning dataset needs to be larger. For future applications in the domain of screening using all ligands available in the Protein Data Bank, a pre-filtering on the basis of simple pocket descriptors (like volume or size) could further accelerate the *sup-CK *method. Future application of the method proposed could include identification of new ligands for protein pockets according to those known for the most similar pockets. This is of interest in the context of identification of drug precursors or of side effects prediction.

## Conclusion

we have developed a new method to measure the similarity between protein binding sites. In this method, binding pockets are described as clouds of points in the 3D space, each point corresponding to an atom. These points may bare additional labels representing various characteristics such as atom partial charges, atom types, or other atomic features. The proposed method showed good performance in the classification of binding pockets according to their respective ligands. It relies on the search for the best global superposition of clouds of atoms, which confers robustness with respect to binding site definition or variations in ligand conformation. This method may be used to compare any type of binding sites in the 3D space, even in absence of overall sequence or structure similarity between their corresponding proteins.

## Authors' contributions

BH and VS prepared the benchmark datasets, performed data processing and interpreted results. MZ and JPV developed the sup-CK algorithm. MZ implemented the methods and performed computational experiments. All authors contributed to the redaction. All authors read and approved the final manuscript.

## Supplementary Material

Additional file 1 **Text file containing the name of all added PDB and ligands for the extension of the Kahraman dataset**. Click here for file

Additional file 2 **Pdf file containing a table describing all proteins used in the Homogeneous dataset**. (PDB name, EC number, ID Uniprot, protein classification, chain, Ligand) Click here for file
