# Supplementary material for: A new protein binding pocket similarity measure based on comparison of clouds of atoms in 3D: application to ligand prediction
Source: BMC Bioinformatics. 2010 Feb 22;11:99. doi: 10.1186/1471-2105-11-99 (PMC2838872; doi:10.1186/1471-2105-11-99)
Supplement: Additional file 2 — Pdf file containing a table describing all proteins used in the Homogeneous dataset. (PDB name, EC number, ID Uniprot, protein classification, chain, Ligand) [file 1471-2105-11-99-S2.PDF]

Homogeneous Dataset

pdb name	EC number	ID Uniprot	protein classification	protein chain	ligand
1a0g	2.6.1.2	P19938	transferase	A	PMP
1a0t	—	P22340	outer membrane protein	P	SUC
1a8i	2.4.1.1	P00489	glycogen phosphorylase	A	LLP
1aia	2.6.1.1	P00509	transferase(aminotransferase)	A	PMP
1aij	—	P0C0Y9	photosynthetic reaction center	M	LDA
1ar1	1.9.3.1	P01636	complex (oxidoreductase/antibody)	B	LDA
1aua	—	P24280	phospholipid-binding protein	A	BOG
1ax4	4.1.99.-	P28796	tryptophan biosynthesis	A	LLP
1b4w	3.1.1.4	O42187	hydrolase	A	BOG
1b56	—	Q01469	lipid-binding	A	PLM
1bjw	2.6.1.1	Q56232	aminotransferase	B	LLP
1bw0	2.6.1.5	P33447	transferase	A	LLP
1c8u	3.1.2.-	P0AGG2	hydrolase	A	LDA
1cl1	4.4.1.8	P06721	methionine biosynthesis	A	LLP
1cmc	—	P0A8U6	dna-binding regulatory protein	B	SAM
1cs1	4.2.99.	P00935	lyase	A	LLP
1d7k	4.1.1.1	P11926	lyase	A	LLP
1dbt	4.1.1.2	P25971	lyase	A	U5P
1dug	2.5.1.1	P08515	blood clotting	B	GSH
1dxr	—	P06010	photosynthetic reaction center	M	LDA
1eem	—	P78417	transferase	A	GSH
1eh5	3.1.2.2	P45478	hydrolase	A	PLM
1eiz	2.1.1.-	P0C0R7	transferase	A	SAM
1f7s	—	Q39250	plant protein	A	LDA
1fg7	2.6.1.9	P06986	transferase	A	PMP
1fgx	2.4.1.3	P08037	transferase	B	U5P
1fw1	2.5.1.1	O43708	isomerase/transferase	A	GSH
1fx8	—	P0AER0	membrane protein	A	BOG
1g8i	—	P62166	metal binding protein	A	1PE
1g8o	2.4.1.1	P14769	transferase	A	U5P
1hmy	2.1.1.3	P05102	transferase(methyltransferase)	A	SAM
1i5e	2.4.2.9	P70881	transferase	A	U5P
1i78	3.4.21.	P09169	hydrolase	B	BOG
1i9g	—	O33253	transferase	A	SAM
1iug	—	Q5SKR1	transferase	A	LLP
1iyh	5.3.99.	O60760	isomerase	A	GSH
1j04	2.6.1.4	P21549	transferase	A	LLP
1jg8	4.1.2.5	Q9X266	lyase	A	LLP
1jgi	2.4.1.4	Q9ZEU2	transferase	A	SUC
1jj0	3.2.1.1	P00698	hydrolase	A	SUC
1jlv	2.5.1.1	Q7KIF2	transferase	A	GSH
1k87	1.5.99.	P09546	oxidoreductase	A	1PE
1k8q	3.1.1.3	P80035	hydrolase	A	BOG
1kmo	—	P13036	membrane protein	A	LDA
1kta	2.6.1.4	O15382	transferase	A	PMP

Continued on next page

pdb name	EC number	ID Uniprot	protein classification	protein chain	ligand
1l0g	3.5.2.6	P00811	hydrolase	A	SUC
1m66	1.1.1.8	P90551	oxidoreductase	A	PLM
1m98	—	P83689	unknown function	A	SUC
1mdo	—	Q8ZNF3	transferase	A	PMP
1mgp	—	Q9X1H9	lipid binding protein	A	PLM
1msk	2.1.1.1	P13009	methyltransferase	A	SAM
1nt2	—	O28191	rna binding protein	A	SAM
1nw3	—	Q8TEK3	transferase	A	SAM
1o57	—	P37551	dna binding protein	C	1PE
1o6u	—	O76054	transferase	A	PLM
1ojd	1.4.3.4	P27338	oxidoreductase	A	LDA
1p91	2.1.1.5	P36999	transferase	A	SAM
1pq2	1.14.14	P10632	oxidoreductase	B	PLM
1pt2	2.4.1.1	P05655	transferase	A	SUC
1q0r	—	Q54528	hydrolase	A	1PE
1qzz	—	Q54527	transferase	A	SAM
1r30	2.8.1.6	P12996	transferase	A	SAM
1r4w	2.5.1.1	P24473	transferase	A	GSH
1s7g	3.5.1.-	O30124	transcription	A	1PE
1sz7	—	O43617	transport protein	A	PLM
1thq	—	P37001	transferase	A	LDA
1tj4	3.1.3.2	P74325	hydrolase	A	SUC
1uc2	—	O59245	unknown function	A	SUC
1umx	—	P0C0Y9	photosynthetic reaction center	H	LDA
1uu1	2.6.1.9	Q9X0D0	transferase	A	PMP
1w2t	3.2.1.2	O33833	hydrolase	A	SUC
1wlj	3.1.-.-	Q96AZ6	hydrolase	A	U5P
1xkw	—	P42512	membrane protein	A	LDA
1y10	4.6.1.1	Q11055	lyase	B	1PE
1y1a	—	Q99828	metal binding protein	B	GSH
1ylj	3.5.2.6	Q9L5C8	hydrolase	A	SUC
1zc9	4.1.1.6	P16932	lyase	A	PMP
1zx8	—	Q9X187	unknown function	C	1PE
2b56	—	Q86MV5	transferase/rna binding protein	A	U5P
2bln	2.1.1.2	P77398	transferase	A	U5P
2bmu	2.7.4.-	Q8U122	transferase	A	U5P
2byn	—	Q8WSF8	receptor	B	1PE
2c37	3.1.13.	Q9UXC2	hydrolase	A	U5P
2c81	2.6.1.-	Q8G8Y2	transferase	A	PMP
2cjg	—	P63509	transferase	A	PMP
2czv	3.1.26.	O59150	hydrolase	C	BOG
2e7u	5.4.3.8	Q5SJS4	isomerase	A	PMP
2fik	—	P11609	immune system	A	PLM
2fls	—	Q9NS18	oxidoreductase	A	GSH
2haw	3.6.1.1	P37487	hydrolase	B	1PE
2hd0	3.4.22.	Q9ELS8	hydrolase	E	BOG
2idb	4.1.1.-	P0AAB4	lyase	A	1PE
2imd	2.5.1.1	Q51948	transferase	A	GSH
2iu8	2.3.1.-	O84245	transferase	B	PLM
2j4j	2.7.4.2	Q97ZE2	transferase	A	U5P
2nwl	—	O59010	transport protein	A	PLM
2p4b	—	P0AFX9	signaling protein	B	BOG
2pbj	5.3.99.-	Q9N0A4	lyase	A	GSH
2z73	—	P31356	membrane protein	A	BOG

Continued on next page

pdb name	EC number	ID Uniprot	protein classification	protein chain	ligand
3b6h	5.3.99.-	Q16647	isomerase	A	BOG
